# COVID-19 Therapeutic Options Under Investigation

**DOI:** 10.3389/fphar.2020.01196

**Published:** 2020-08-06

**Authors:** Malak Kaddoura, Malak AlIbrahim, Ghina Hijazi, Nadia Soudani, Amani Audi, Habib Alkalamouni, Salame Haddad, Ali Eid, Hassan Zaraket

**Affiliations:** ^1^ Department of Experimental Pathology, Immunology & Microbiology, Faculty of Medicine, American University of Beirut, Beirut, Lebanon; ^2^ Center for Infectious Disease Research, Faculty of Medicine, American University of Beirut, Beirut, Lebanon; ^3^ Department of Pharmacology and Toxicology, Faculty of Medicine, American University of Beirut, Beirut, Lebanon

**Keywords:** SARS-CoV-2, COVID-19, antivirals, adjunctive therapy, therapeutics

## Abstract

Since its emergence in China in December 2019, COVID-19 has quickly spread around the globe causing a pandemic. Vaccination or the development of herd immunity seems the only way to slow down the spread of the virus; however, both are not achievable in the near future. Therefore, effective treatments to mitigate the burden of this pandemic and reduce mortality rates are urgently needed. Preclinical and clinical studies of potential antiviral and immunomodulatory compounds and molecules to identify safe and efficacious therapeutics for COVID-19 are ongoing. Two compounds, remdesivir, and dexamethasone have been so far shown to reduce COVID-19–associated death. Here, we provide a review of the potential therapeutic agents being considered for the treatment and management of COVID-19 patients.

## Introduction

The emergence of viral diseases causing respiratory illnesses, and thus threatening human health continues to be a critical threat to public health and the economy. During the last two decades, four major viral outbreaks were recorded. These are the severe acute respiratory syndrome coronavirus “SARS-CoV-1” (Zhong et al., 2003), influenza A/H1N1 (H1N1pdm09) (Dotis and Roilides, 2009), Zika virus (Fauci and Morens, 2016) and the Middle East respiratory syndrome coronavirus “MERS-CoV” (Zaki et al., 2012). H1N1pdm09 and Zika viruses became pandemic, SARS-CoV-1 vanished, while MERS-CoV continues to cause infections but is largely contained.

In December 2019, an outbreak of pneumonia of unknown etiology was reported in Wuhan, China (World Health Organization). A novel coronavirus (CoV), initially named 2019-nCoV, was isolated and identified as the culprit (Zhu et al., 2020). On February 11, 2020, the illness associated with this viral infection was given the name COVID-19 “COronaVIrus Disease 2019” by the World Health Organization (WHO). Owing to its similarity to SARS-like CoVs, the 2019-nCoV has been renamed by the International Committee on Taxonomy of Viruses (ICTV) as SARS-CoV-2. On March 11, 2020, WHO Director-General Tedros Adhanom Ghebreyesus declared COVID-19 a global pandemic (World Health Organization, 2020b).

SARS-CoV-2 is a highly contagious virus that continues to spread at an unprecedented rate; globally confirmed cases exceeded 16 million of which more than 650 000 resulted in death, as of the 29th of July (World Health Organization, 2020c).

The basic reproduction number of COVID-19 has been estimated at 2 to 2.5 (World Health Organization, 2020a). SARS-CoV-2 has a median incubation period of 5.1 days and 97.5% of symptomatic patients will develop symptoms within 11.5 days. Moreover, 1% of patients will become symptomatic after 14 days of exposure (Lauer et al., 2020). Patients infected with SARS-CoV-2 mainly experience mild symptoms including fever, cough, fatigue, loss of appetite, difficulty breathing, and muscle pain (Singhal, 2020). Digestive symptoms, such as loss of appetite, nausea, vomiting, diarrhea, and abdominal pain, have also been reported among COVID-19 patients (Pan et al., 2020). Other symptoms such as anosmia or ageusia were also reported among COVID-19 patients (Giacomelli et al., 2020). Skin manifestations including erythematous rash urticaria and pruritus lesions, particularly among young individuals have been also associated with COVID-19 (Recalcati, 2020; Tao et al., 2020). Unfortunately, SARS-CoV-2 infection might result in more severe complications including severe pneumonia and acute respiratory distress syndrome, and may also lead to death (Wang L. et al., 2020).

CoVs are enveloped, positive-sense, single-stranded RNA viruses (~30 kb) with a 5′-cap structure and 3′-poly-A tail. Their name originates from the Latin word *coronam* which means crown, representing their crown-like morphology under the microscope. They belong to *the Orthocoronavirinae* subfamily (*Coronaviridae* family, *Nidovirales* order), which includes four genera; *Alphacoronavirus* and *Betacoronavirus* (infecting only mammals), *Gammacoronavirus* and *Deltacoronavirus (*mainly infecting birds). The *Betacoronavirus* genus to which SARS-CoV-2 belongs is divided into five lineages (Woo et al., 2012).

To date, seven human coronaviruses (HCoVs) have been detected. The common HCoVs [HCoV-OC43 and HCoV-HKU1 (betaCoVs, A lineage), HCoV-229E, and HCoV-NL63 (alphaCoVs)] usually cause mild self-limiting upper respiratory tract illnesses in immunocompetent individuals. But they can result in lower respiratory tract symptoms in immunocompromised patients and the elderly (Trombetta et al., 2016). The highly pathogenic SARS-CoV-1, SARS-CoV-2 (betaCoVs of the B lineage), and MERS-CoV (betaCoV of the C lineage) cause mild to severe pulmonary and extra-pulmonary disease (Su et al., 2016; Chen Y. et al., 2020).

SARS-CoV-2 genome is made up of ~30 kilobases (Chen L. et al., 2020; Zhou P. et al., 2020), and shares 96.2% identity with bat CoV isolate RaTG13, suggesting a bat origin for this virus (Lu et al., 2020; Zhou P. et al., 2020; Zhu et al., 2020). Aside from RaTG13, the Pangolin-CoV was very closely related to SARS-CoV-2, sharing 91% nucleotide similarity thereby suspecting that pangolin might be the intermediate host (22,23). Moreover, SARS-CoV-2 has about 79% and 50% nucleotide sequence identity with SARS-CoV-1 and MERS-CoV, respectively (Zhou P. et al., 2020). Like other betacoronaviruses, SARS-CoV-2 has a 5′ long nonstructural polyprotein (ORF1ab), and four major structural proteins (spike glycoprotein (S), envelope protein (E), matrix protein (M) and nucleocapsid protein(N)) (Phan, 2020). Importantly, genetic analysis of SARS-CoV-2 from different countries reveals that the virus has diversified into several genetic clades (www.nextstrain.org/ncov/global).

There is no doubt that the development of vaccines or antiviral drugs is critical for mitigating the burden of the COVID-19 pandemic (Petherick, 2020). Ongoing preclinical and clinical studies aim to investigate the safety and efficacy of existing molecules or repurposed drugs in the treatment and management of COVID-19. This review focuses on the potential antivirals and adjunctive therapies that are being investigated as therapeutic tools in the fight against COVID-19.

### SARS-Like Coronaviruses Lifecycle

SARS-CoV-2 virions are studded with spike (S) glycoproteins. The S protein serves as a primary target for the host-generated antibody response, entry inhibitors, and vaccines due to its role in viral attachment, fusion, and entry (Du et al., 2009; Yuan et al., 2020). It mediates viral entry into host cells by first binding to the host cell *via* its receptor-binding domain (RBD) in the S1 subunit and then mediating the fusion between the viral and host membranes through the S2 subunit (Liu et al., 2004; Li et al., 2005). Similar to SARS-CoV-1, SARS-CoV-2 also recognizes angiotensin-converting enzyme 2 (ACE2) as its host receptor (Li et al., 2003).

Interestingly, SARS-CoV-1 RBD-specific polyclonal antibodies could cross-neutralize SARS-CoV-1 and SARS-CoV-2 pseudovirus infection, by blocking their entry into human ACE2 (hACE2)-expressing cells, suggesting the potential to develop RBD-based vaccine for prevention of infection by SARS-CoV-2 (Tai et al., 2020). Following receptor binding, the virus must gain access to the host cell cytosol. This is accomplished by acid-dependent proteolytic cleavage of S protein by a lysosomal cathepsin L, followed by fusion of the viral and cellular membranes (Wang et al., 2008). This proteolytic cleavage occurs at two sites, the first at the S1/S2 site and is important for separating the RBD and fusion domains of the protein; and the second at the S2′ site and is important for exposing the fusion peptide (Belouzard et al., 2009). A recent study showed that TMPRSS2-expressing Vero E6 cell line displays enhanced SARS-CoV-2 infection compared to parenteral Vero cells (Matsuyama et al., 2020). Inhibiting TMPRSS2 serine protease blocked the entry of VSV-SARS-2-S pseudotypes, supporting the important role of TMPRSS2 in SARS-CoV-2 entry. Fusion occurs within acidified endosomes, then, releasing the viral genome into the cytoplasm (Fehr and Perlman, 2015). The next step in the virus lifecycle is the translation of the replicase genes from the virion genomic RNA. The replicase gene encodes two large ORFs, ORF1a and ORF1b, which express two co-terminal polyproteins, pp1a and pp1ab (Baranov et al., 2005). The main protease M^pro^, also called 3CL^pro^, is considered a highly promising target to treat different coronaviruses strains (Zhang L. et al., 2020). This protease, in addition to the papain-like protease(s), is crucial for polyprotein processing (Hilgenfeld, 2014; Zhang L. et al., 2020). Therefore, blocking the viral replication is possible by targeting this enzyme, and its inhibition is not expected to be harmful because there are no identified human proteases that possess the same cleavage specificity.

**Figure 1:**
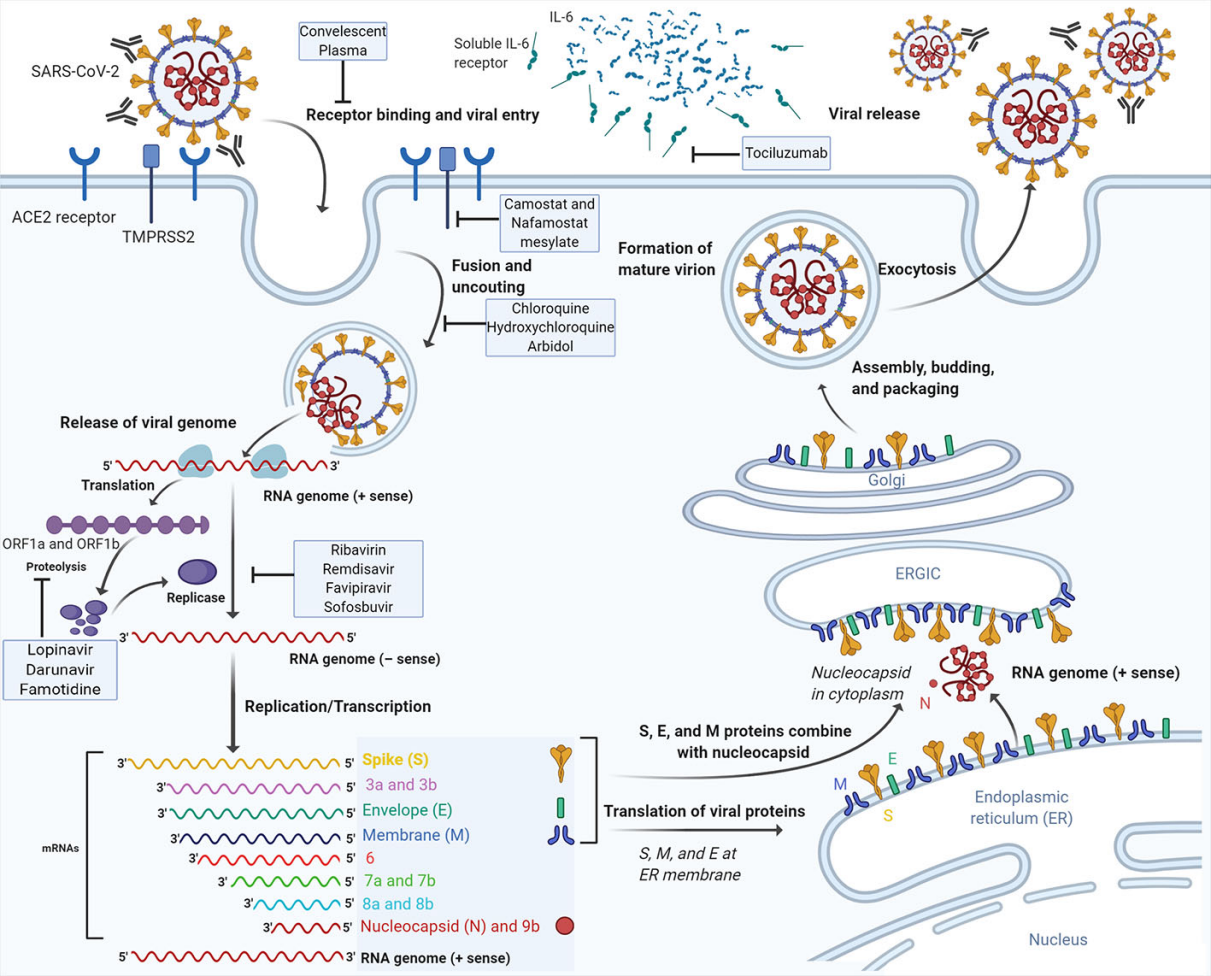
The life cycle of SARS-CoV-2 and the mode of action of potential therapeutic molecules. SARS-CoV-2 enters target cells through an endosomal pathway. The S protein of the virus binds to cellular receptor ACE2. Following the entry of the virus into the host cell, the viral RNA is unveiled in the cytoplasm. ORF1a and ORF1ab are translated to produce pp1a and pp1ab polyproteins, which are cleaved by the proteases that are encoded by ORF1a to yield non-structural proteins that form the RNA replicase-transcriptase complex. The polymerase produces a series of subgenomic mRNAs by discontinuous transcription and finally translated into relevant viral proteins. Viral nucleocapsids are assembled from genomic RNA and N protein in the cytoplasm, followed by budding into the lumen of the ERGIC. Virions are then released from the infected cell through exocytosis. ACE2, angiotensin-converting enzyme 2; ER, endoplasmic reticulum; ERGIC, ER-Golgi intermediate compartment. Drugs with potential anti-SARS-CoV-2 activity are depicted on the corresponding stage of the virus life cycle which they are thought to interfere with.

The next step in the virus lifecycle, after the translation and assembly of the viral replicase complexes, is the viral RNA synthesis. This viral RNA synthesis produces both genomic and sub-genomic RNAs. Sub-genomic RNAs serve as mRNAs for the structural and accessory genes. These genes reside downstream of the replicase ORF. Through negative-strand intermediates, genomic and sub-genomic RNAs are generated (Fehr and Perlman, 2015). Following replication and subgenomic RNA synthesis, the viral structural proteins, S, E, and M are translated and inserted into the endoplasmic reticulum (ER). These proteins move along the secretory pathway into the ER–Golgi intermediate compartment (ERGIC) (He et al., 2014). In this intermediate compartment, the mature virions are produced by budding of the viral genomes encapsidated by N protein into its membranes holding the viral structural proteins (de Haan and Rottier, 2005). M and E proteins form the viral envelope and are sufficient for the generation of virus-like particles (VLP). Moreover, N protein was shown to enhance VLP production, proposing that the fusion of encapsidated genomes into the ERGIC complement envelope formation (Siu et al., 2008). At this level, the inclusion of the S protein into virions takes place but is not needed for assembly. However, this step needs the capacity of this protein to traffic to the ERGIC and interact with the M protein. Once the M protein attaches to the nucleocapsid, the assembly will be accomplished (Hurst et al., 2005). Finally, virions are transported to the cell membrane *via* transport vesicles and are then liberated by exocytosis (**Figure 1**).

## Drugs Under Investigation For Treatment And Management of Covid-19

In this review, we classified the various drugs currently under investigation into three categories: virus targeting, host targeting, and adjunctive therapy. The direct-active antivirals represent a class of drugs that exert its activity by directly targeting viral components thus blocking replication. Host-targeting compounds represent a class of drugs that interfere with host factors crucially required for viral infection and replication. While the disease-modulating adjunctive therapy includes molecules that modulate the host immune response to reduce inflammation. Finally, drugs with more than one mode of action were included in the mixed mode of action category. A summary of all drugs being considered for COVID-19 treatment and management is presented in **Table 1**. In addition, data available from clinical trials were summarized in **Table 2**.

**Table 1:** Potential SARS-CoV-2 therapeutic agents.

Compound/drug	Mechanism of action	*In vitro* studies	Randomized clinical trials	
			Ongoing	Complete
***Virus targeting agents***				
Remdesivir	Inhibits RNA-dependent RNA polymerase	Yes EC50 = 0.77 - 23.15 μM Vero E6 cells	Yes	Yes
Lopinavir/Ritonavir (Kaletra)	Protease inhibitor with a CYP3A4 inhibitory activity	Yes EC50 = 26.63 μM Vero E6 cells	Yes	Yes
Favipiravir	Inhibits RNA-dependent RNA polymerase	No	Yes	Yes
Ribavirin	Blocks viral RNA synthesis and viral mRNA capping	No	Yes	Yes
Famotidine	Histamine-2 (H2) receptor antagonist 3CL^pro^ targeting	No	Yes	No
EIED 2801	Impairs viral replication by incorporating into the genome of the newly formed virions	Yes EC50 = 0.3 μM Vero E6	Yes	No
Oseltamivir	Inhibits neuraminidase enzyme	No	Yes	No
Sofosbuvir	Inhibits RNA-dependent RNA polymerase	No	No	No
Penciclovir	Inhibits viral DNA polymerase	Yes EC50 = 95.96 μM Vero E6	No	No
Azvudine	Inhibiting nucleoside reverse-transcriptase	No	Yes	No
Triazavirin	Inhibits RNA synthesis	No	Yes	No
ACE2 decoy receptor	ACE2 antagonist	Yes 6–100 µg/ml	Yes	No
***Host-targeting agents***				
Azithromycin	Stimulates the interferon pathway Interferes with virus internalization	Yes	Yes	No
Ivermectin	Impairs nuclear import by interacting with importin (IMP) α/β1 heterodimer	Yes EC50 = 2 µM Vero-hSLAM cells	Yes	No
Nafamostat and Camostat	Inhibits fusion-activation of the virus through inhibition of the host protease (TMPRSS2)	Yes EC50 = 22.50 µM Vero E6 cells	Yes	No
Teicoplanin	Suppresses the entry by blocking the activity of cathepsin L in the late endosome/lysosome	Yes EC50 = 1.66 µM Vero E6 cells	No	No
Nitazoxanide	Blocks viral entry and replication Inhibits the production of pro-inflammatory cytokines	Yes EC50 = 2.12 μM Vero E6 cells	Yes	No
***Drugs with mixed action***				
Umifenovir	-Inhibits membrane fusion through interacting with the viral glycoproteins - Elevate endosomal pH	Yes EC50 = 4.11 μM Vero E6 cells	Yes	No
Chloroquine phosphate and hydroxychloroquine	-Hinders the auto-immune response -Impairing ACE2 terminal glycosylation-Increasing the endosomal pH	Yes CQ EC50 = 1.13–5.74 μM HCQ EC50 = 0.72 μM Vero E6 cells	Yes	Yes
***Adjunctive therapy***				
Immunomodulatory agents				
Fingolimod	Targets sphingosine-1-phosphate (S1P) receptors and alters the signaling of the S1P pathway	No	Yes	No
Thymosin α1	Triggers lymphocyte maturation Enhances T cell activation	No	Yes	No
Tocilizumab	Recombinant anti-human interleukin-6 receptor (IL-6R) monoclonal antibody	No	Yes	No
Bevacizumab	Humanized monoclonal antibody against the angiogenic vascular endothelial growth factor (VEGF)	No	Yes	No
Colchicine	Down-regulates multiple inflammatory pathways through tubulin disruption Inhibits microtubule-dependent chemotaxis of neutrophils, generation of leukotrienes and cytokines, phagocytosis, and the (TNF-α)-induced NF-κB pathway	No	Yes	Yes
Methylprednisolone	Anti-inflammatory properties at high doses	Not applicable	Yes	No
Dexamethasone	Anti-inflammatory properties	Not applicable	Yes	Yes
Convalescent plasma	Provides passive immunization	Not applicable	Yes	No

**Table 2:** Summary of COVID-19 therapeutics that have completed clinical trials.

Ref	Compound	Route	Dosing regimen	Study design	Evidence type^#^	Summary
(Wang Y. et al., 2020)	Remdesivir	Intravenous	200 mg on day 1 followed by 100 mg on days 2–10 in single daily infusions	Randomized, double-blind, placebo-controlled, multicenter trial	Moderate	Aim: To evaluate the safety and efficacy of remdesivir in hospitalized adults infected with SARS-CoV-2. Key findings: No significant difference in improvement time. Adverse effects: Same as placebo.
(Beigel et al., 2020)	Remdesivir	Intravenous	200 mg on the first day followed by a 100-mg once daily maintenance dose for up to a 10 days	Multicenter, adaptive, randomized, double-blind, placebo-controlled trial	Strong	Aim: To evaluate the safety and efficacy of remdesivir in hospitalized adults infected with SARS-CoV-2. Key findings: Patients receiving remdesivir recovered faster than those treated with placebo (median recovery time of 11 days and 15 days, respectively). The risk of death by 14 days was less in the remdesivir group compared with the placebo one; 7.1% and 11.9% respectively. Adverse effects: Same as placebo.
(Cao et al., 2020)	Lopinavir/ritonavir	oral	400 mg/100 mg twice daily for 14 days	Randomized, controlled, open-label trial	Moderate	Aim: To study the efficacy of lopinavir/ritonavir in hospitalized adult patients with severe COVID-19. Key findings: No clinical benefit. Adverse effects: Greater than placebo primarily gastrointestinal side effects.
(Chen C. et al., 2020)	Favipiravir or umifenovir	Oral	Favipiravir 1,600 mg twice daily 1st day then 600 mg twice daily for 10 days vs. umifenovir 200 mg three times daily for 10 days	Prospective, randomized, controlled, open-label.	Moderate	Aim: To evaluate the clinical efficacy and safety of favipiravir versus umifenovir as a treatment for COVID-19. Key findings: No improvement in clinical recovery at day 7. Improved the time to relief for pyrexia and cough compared to umifenovir. Adverse effects: raised serum uric acid was more frequently observed in favipiravir group.
(Cai et al., 2020)	Favipiravir	Oral	Favipiravir 1,600 mg twice daily 1^st^ day then 600 mg twice daily for 14 days + 5mIU of IFN-α twice daily vs. lopinavir/ritonavir 400 mg/100 mg twice daily + 5 mIU of IFN-α twice daily for 14 days	Open-label nonrandomized- comparative controlled study	Weak	Aim: To examine the efficacy of favipiravir versus lopinavir/ritonavir for the treatment of COVID-19. Key Findings: FPV showed better therapeutic responses than LPV/RTV. Adverse effects: Generally mild but less common in the favipiravir treated group.
(Hung et al., 2020)	Ribavirin	Oral	400mg twice daily for 14 days	Prospective, randomized, controlled, open-label trial	Moderate	Aim: To evaluate the safety and efficacy of IFN-ß-1b, lopinavir/ritonavir, and ribavirin combination. Key findings: Recovery was accelerated, viral load was suppressed, hospitalization was shortened and mortality was reduced after the combination of lopinavir/ritonavir, ribavirin, and IFN-ß-1b compared with to lopinavir/ritonavir alone (control). Adverse effects: Same as placebo.
(Li Y. et al., 2020)	Umifenovir or lopinavir/ritinovir	Oral	Lopinavir 200 mg plus ritonavir 500mg twice daily for 7–14 days vs. umifenovir 200 mg three times daily for 7–14 days	Open-label randomized controlled trial	Moderate	Aim: To explore the efficacy and safety of lopinavir/ritonavir or umifenovir monotherapy for the treatment of patients hospitalized with mild/moderate COVID-19. Key findings: lopinavir/ritonavir or umifenovir monotherapy offered minimal added benefit compared to standard of care. Adverse events: Greater than control with diarrhea being most common.
(Gautret et al., 2020)	HCQ/AZM	Oral	HCQ 200 mg three times daily for 10 days Azithromycin 500 mg on day 1 followed by 250 mg daily for four consecutive days	Open-label non-randomized clinical trial	Weak	Aim: To investigate the efficacy of HCQ in COVID-19 patients and the role of adding AZM Key findings: Significant reduction in viral load in patients receiving HCQ alone. 100% recovery in patients receiving a combination of AZM and HCQ. Adverse effects: Not described.
(Boulware et al., 2020)	HCQ	Oral	800 mg as a first dose, followed by 600 mg after 6 to 8 h, then 600 mg daily for 4 days	Randomized, double-blind, placebo-controlled trial	Strong	Aim: To assess HCQ as post-exposure (within 4 days of exposure) prophylaxis for COVID-19 Key findings: HCQ did not prevent laboratory confirmed infection or COVID-19 like illness compared to placebo Adverse effects: Greater than placebo but not serious
(Mitjà et al., 2020)	HCQ	Oral	800 mg on the first day, followed by 400 mg once daily for 6 days	Multicenter, open label, randomized controlled trail	Moderate	Aim: To evaluate the efficacy of early administration of HCQ in non-hospitalized adults with mild COVID-19 Key findings: No significant difference in viral load reduction, risk of hospitalization, and clinical recovery compared to standard care. Adverse effects: Same as placebo
(Skipper et al., 2020)	HCQ	Oral	800 mg as a first dose followed by 600 mg after 6 to 8 h, then 600 mg daily for 4 days	Randomized, double blinded, placebo controlled trial	Strong	Aim: To assess the efficacy of HCQ in decreasing the disease severity in adult outpatients with early, mild COVID-19 Key findings: No significant decrease in the severity of symptoms compared to placebo Adverse effects: Greater than placebo- none were serious
(Deftereos et al., 2020)	Colchicine	Oral	1.5-mg loading dose followed by 0.5 mg after 60 min and maintenance doses of 0.5 mg twice daily for 3 weeks	Prospective, open-label, randomized clinical trial	Moderate	Aim: To evaluate the effect of treatment with colchicine on cardiac and inflammatory biomarkers and clinical outcomes in COVID-19 hospitalized patients Key findings: Improved time to clinical deterioration. Adverse effects: Similar to the control group except for diarrhea being more frequent with colchicine.
(The RECOVERY Collaborative Group, 2020)	Dexamethasone	Oral or intravenous	6 mg once daily for 10 days	Randomized, controlled, open-label, adaptive, platform trial	Moderate	Aim: To evaluate dexamethasone in hospitalized COVID-19 patients. Key findings: Dexamethasone reduced mortality among those receiving invasive mechanical ventilation or oxygen but not among milder cases. Adverse effects: Not described.
(Joyner et al., 2020)	Convalescent Plasma	Intravenous	200 – 500 mL	Non-randomized	Weak	Aim: To investigate the safety of convalescent plasma treatment in hospitalized COVID-19 patients Key findings: Transfusion of convalescent plasma is safe. Adverse effects: Frequency of <1% of all transfusions. Include transfusion-associated circulatory overload (TACO), transfusion-related acute lung injury (TRALI), severe allergic transfusion reaction, and death.
(Li L. et al., 2020)	Convalescent Plasma	Intravenous	4 to 13 ml/kg	Open-label, multicenter, randomized clinical trial	Moderate	Aim: To evaluate the efficacy and adverse effects of convalescent plasma therapy for patients with COVID-19. Key findings: No statistically significant difference in clinical improvement within 28 days. Negative viral PCR conversion rate was significantly higher in the convalescent plasma group. Adverse effects: Reported in two patients. Included chills and rashes in one patient and shortness of breath, cyanosis, and severe dyspnea within 6 h of transfusion in another one.

^#^Non-randomized trials were considered as having weak evidence, randomized and open-labelled studies were classified as moderate, and randomized, double-blinded studies were considered strong evidence. Randomized, double blinded studies that could not complete enrollment (underpowered) were downgraded to moderate.

### Virus Targeting Agents

#### Remdesivir

Remdesivir (GS-5734) is an investigational monophosphoramidate prodrug of an adenine analog that was originally tested against the Ebola virus in rhesus monkeys (Warren et al., 2016). Biochemical analysis revealed that remdesivir blocks the replication of the Ebola virus by inhibiting its RNA-dependent RNA polymerase (Tchesnokov et al., 2019). However, based on an interim analysis of the PALM trial, remdesivir was dropped from clinical trials after showing inferior outcomes to REGN-EB3, a cocktail of three monoclonal antibodies (Mulangu et al., 2019). Besides the Ebola virus, remdesivir showed *in vitro* and *in vivo* inhibitory activity against the Nipah virus (Lo et al., 2017; Lo et al., 2019). In 2017, *in vitro* studies (using primary human airway epithelial cells) revealed that remdesivir also possesses a broad-spectrum antiviral activity against epidemic and zoonotic CoVs including MERS-CoV and SARS-CoV-1 (Sheahan et al., 2017). This anti-CoV-activity is achieved through inhibiting the RNA polymerase and early termination of transcription (Agostini et al., 2018). Moreover, *in vivo* investigations, using a SARS-CoV-1 mouse model revealed that prophylactic and therapeutic administration of remdesivir resulted in a significant decrease in lung viral titers and mitigation of the disease (Sheahan et al., 2017). Remdesivir exhibits superior *in vitro* (Calu-3 cells) antiviral activity to lopinavir/ritonavir-alone or in combination with interferon (INF-β) against MERS-CoV. Besides, therapeutic administration of remdesivir acute lung injury in mice but without reducing viral loads (Sheahan et al., 2020a). Remdesivir was also shown to possess prophylactic and therapeutic efficacy against SARS-CoV-2 in rhesus macaque models (de Wit et al., 2020).

Owing to the promising antiviral activity of remdesivir against SARS-CoV-1 and MERS-CoV, it was considered as a potential antiviral drug against SARS-CoV-2. *In vitro* studies, using Vero E6 cells, revealed a significant reduction in SARS-CoV-2 yield with a 50% effective concentration (EC50) of 0.77 – 23.15 μM (Choy et al., 2020; Wang M. et al., 2020). Moreover, *in silico* studies showed that remdesivir could tightly bind SARS-CoV-2 RdRps (Elfiky, 2020b). The *in vivo* efficacy of remdesivir was evaluated rhesus macaques infected with SARS-CoV-2 showing clear clinical benefit with a reduction in viral load and lung damage in treated animals (Williamson et al., 2020, 2). Notably, the treatment of these animals was initiated 12 h after SARS-CoV-2 inoculation. It remains important to assess whether the clinical benefit can be maintained if treatment was delayed. Early data from the compassionate use of remdesivir revealed that the treatment was associated with clinical improvement in 68% of hospitalized patients with severe COVID-19 (Grein et al., 2020, 19). However, this study was limited to being observational and non-randomized. A randomized double-blinded multicenter trial that was conducted among adult patients infected with SARS-CoV-2 showed that the administration of remdesivir did not possess any significant therapeutic advantage. Even though the early administration of remdesivir shortened the time needed for clinical improvement, it did not reach statistical significance due to the small sample size which led to the termination of the study (Wang Y. et al., 2020). Preliminary data of a randomized trial conducted on 1063 COVID-19 patients showed that the use of remdesivir significantly sped up the recovery time by four days (11 days vs 15 days for placebo; relative risk 1.04 [0.91–1.18]; p <0.001) and reduced the death rate by 14 days (7.1% vs 11.9% for placebo; p=0.059) (Beigel et al., 2020). Another randomized, open-label, phase 3 trial found that hospitalized patients who did not require mechanical ventilation and received a shorter, 5-day regimen, experienced similar clinical improvement as patients who received a 10-day regimen (Goldman et al., 2020, 19). In this context, on May 1, 2020, the Food and Drug Administration (FDA) has approved the emergency use of remdesivir in SARS-CoV-2–infected patients (Food and Drug Adminstration, 2020). A very recent GILEAD press release report presented new findings regarding remdesivir; a comparative analysis of Phase 3 SIMPLE-Severe trial and a real-world retrospective cohort of patients with severe SARS-CoV-2 infection revealed that remdesivir resulted in better improvement and diminished death rate by 62% compared to standard care (GILEAD, 2020). Simultaneously, several clinical trials are ongoing to assess the safety and efficacy of remdesivir, including two randomized ones.

#### Lopinavir/Ritonavir

Lopinavir/ritonavir combination is an FDA-approved drug indicated for the treatment of HIV-1. Lopinavir is an anti-retroviral protease inhibitor that undergoes extensive hepatic metabolism. In this context, ritonavir, a protease inhibitor with a CYP3A4 inhibitory activity, is used in combination with lopinavir to increase its bioavailability and boost its antiviral activity (Food and Drug Administration; De Clercq, 2009). *In vitro*, lopinavir significantly inhibits the replication of MERS-CoV, SARS-CoV-1, and HCoV-229E (Chen et al., 2004; Chu et al., 2004; de Wilde et al., 2014). Treatment of MERS-CoV-infected common marmoset with lopinavir/ritonavir resulted in improved clinical outcomes and reduced lung viral loads (Chan et al., 2015). Clinical trials investigating the efficacy of lopinavir/ritonavir, combined with INF- β 1b against MERS-CoV (NCT02845843) is ongoing.

With the emergence of SARS-CoV-2, lopinavir/ritonavir was quickly considered as one of the potential treatment options. Lopinavir inhibits the replication of SARS-CoV-2 virus in Vero E6 cells with an EC50 of 26.63 μM (Choy et al., 2020). Kang et al. further showed that lopinavir/ritonavir possesses significant inhibitory activity against SARS-CoV-2 *in vitro* at concentrations (7/1.75 μg/mL) equivalent to their steady-state plasma levels (Kang et al., 2020). Clinically, a retrospective analysis of hospitalized COVID-19 patients showed that only early administration (≤10 days from disease onset) of lopinavir/ritonavir was associated with shorter duration of virus shedding (Yan et al., 2020). These observations were not supported by the stronger evidence originating from randomized clinical trials that showed a lack of benefit for lopinavir/ritonavir treatment. A randomized, controlled, open-label trial in China showed that a twice-daily two-week regimen of lopinavir/ritonavir for hospitalized adult patients with severe COVID-19 was not associated with significant benefits compared to the control one (standard care only) (Cao et al., 2020). In this study, 14% of the patients could not complete the treatment regimen due to adverse events. Consistently, unpublished data from the RECOVERY (Randomized Evaluation of COVid-19 thERapY) trial conducted in the United Kingdom revealed a lack of clinical benefit for lopinavir/ritonavir in patients hospitalized with COVID-19, leading to a decision to halt enrollment in this treatment arm. According to a news release on the RECOVERY Trial website, 1,596 and 3,376 patients were randomized to lopinavir/ritonavir and standard of care alone, respectively. The 28-day mortality was 22.1% for the lopinavir/ritonavir compared to 21.3% for the standard of care (*p* = 0.58) (Horby and Landray, 2020b). Consistently, the WHO solidarity trial announced that it will be discontinuing the lopinavir/ritonavir arm after preliminary analysis revealed little or no reduction in mortality (World Health Organization, 2020d). The data from both the RECOVERY and Solidarity trials have not been fully published to enable a complete assessment of the findings. Randomized trials are also ongoing to investigate the clinical efficacy and safety of darunavir, second-generation retroviral protease inhibitor in combination with a pharmacokinetic enhancer (cobicistat) in COVID-19 patients (Canadian Agency for Drugs and Technologies in Health, 2015).

#### Favipiravir

Favipiravir is a potent antiviral drug licensed in Japan in 2014 for novel influenza strains (Hayden and Shindo, 2019). Favipiravir selectively and potently inhibits the RNA-dependent RNA polymerase (RdRp) of RNA viruses. It undergoes phosphoribosylation and further phosphorylation intracellularly to become favipiravir ribofuranosyl-5′-triphosphate (favipiravir-RTP). The active favipiravir-RTP act as a terminator of nascent RNA strand elongation by competing with purine nucleosides for RdRp binding (Sangawa et al., 2013). Other studies revealed that favipiravir induces its antiviral activity by acting as a lethal mutagen (Baranovich et al., 2013). It has shown notable efficacy against a broad-spectrum of lethal RNA viruses as the Ebola virus (Guedj et al., 2018), Lassa virus (Rosenke et al., 2018), rabies (Yamada et al., 2016), and severe fever with thrombocytopenia syndrome (Tani et al., 2018). However, in embryo-fetal developmental studies, favipiravir exposure has a risk for teratogenicity and embryotoxicity (Nagata et al., 2015). A preprint report of an open-label randomized clinical trial in Wuhan, China (ChiCTR200030254) concluded that favipiravir significantly improve the clinical recovery rate by seven days compared to umifenovir (Chen C. et al., 2020). Another open-label, comparative, controlled study (ChiCTR2000029600) showed favipiravir exerts a more potent antiviral action against COVID-19 than lopinavir/ritonavir and with greater safety. Favipiravir showed significantly faster viral clearance and a higher improvement rate in chest imaging of treated patients compared to a control group treated with lopinavir/ritonavir (Cai et al., 2020). Several randomized trials were launched in China to test the effectiveness and efficacy of favipiravir either combined with other drugs or as a monotherapy.

#### Ribavirin

Ribavirin is a synthetic guanosine nucleoside that blocks viral RNA synthesis and viral mRNA capping (Sidwell et al., 1972). This prodrug, which is metabolized into nucleoside analogs, was originally tested against the respiratory syncytial virus (RSV) in pediatric patients showing only marginal clinical benefit (Ventre and Randolph, 2004). Besides RSV, ribavirin is primarily indicated for treating hepatitis C and viral hemorrhagic fevers (Te et al., 2007). A recent study reported that SARS-CoV-1 is sensitive to high doses of ribavirin but the effect is cell line-dependent (Tan et al., 2004). Ribavirin displayed an inhibitory effect against MERS-CoV in Vero cells and LLC-MK2 cells but only at high concentrations (Falzarano et al., 2013). Although a number of laboratories have shown ribavirin to be in inhibiting SARS-CoV-1 *in vitro* (Koren et al., 2003; Chen et al., 2004), others failed (Cinatl et al., 2003; Barnard et al., 2004; Tan et al., 2004). Surprisingly, studies in mice revealed that ribavirin given during the first three days of infection increased SARS-CoV-1 lung viral loads and prolonged the duration of infection (Subbarao et al., 2004). In addition to the apparent lack of efficacy in most human studies, the risk of ribavirin-induced anemia along with hypoxia resulted in an increased risk of death in the treated SARS-CoV-1 patients (Barnard et al., 2006a). Thus, ribavirin may not be useful for treating SARS-CoV-1 infection because of its questionable efficacy and known toxicity. In molecular docking studies, ribavirin was predicted to tightly bind to SARS-CoV-2 RdRp (Elfiky, 2020b), but its antiviral activity has not been confirmed *in vitro*. The safety and efficacy of ribavirin combined with lopinavir/ritonavir and IFN-ß-1b were assessed in an open-label, randomized clinical trial (NCT04276688) in Hong Kong for hospitalized adult patients with confirmed SARS-CoV-2 infection (Hung et al., 2020). The triple antiviral combination significantly reduced the time to negative nasopharyngeal swab by an average of 5 days and time to complete alleviation of symptoms by 4 days compared to the lopinavir/ritonavir (control group). However, the study lacked a placebo group and did not include critically ill patients. Also, the lack of a ribavirin and IFN-ß-1b monotherapy groups curtailed the assessment of the clinical benefit of each drug alone and warrants further investigations.

#### Famotidine

Famotidine is an FDA approved histamine-2 (H2) receptor antagonist indicated for suppressing gastric acid secretion (Food and Drug Administration, 1986). Computer-based target-drug screening of SARS-CoV-2 revealed that famotidine targets the 3CL^pro^ and may be a potential therapeutic option for the treatment of COVID-19 (Wu et al., 2020). The clinical impact of famotidine on the prognosis of COVID-19 was accidentally noticed; Janowitz et al demonstrated that a group of 10 SARS-CoV-2–infected patients who received high famotidine doses (240 mg in three divided doses for a median of 11 days) showed significant improvement in symptoms within 24 h of drug administration (Janowitz et al., 2020). Furthermore, a retrospective cohort study revealed that the use of famotidine significantly decreased the mortality rate and diminished the need for mechanical ventilation (Freedberg et al., 2020). In this context, a randomized clinical trial is being conducted to study the efficacy and safety of using a high dose of intravenous famotidine in COVID-19 hospitalized patients.

#### EIDD 2801

EIDD-2801 is an orally bioavailable β-d-N4-hydroxycytidine (NHC)-prodrug with broad-spectrum antiviral activity (Toots et al., 2019). EIDD-2801 is metabolized *in vivo* into NHC (EIDD-1931), a pyrimidine ribonucleoside analog (Toots et al., 2019). The metabolite is incorporated into the genome of the newly formed virions resulting in the accumulation of numerous mutations that impair viral replication (Urakova et al., 2018). NHC was shown to have antiviral activity against several RNA viruses including influenza virus, RSV, chikungunya virus, Venezuelan equine encephalitis virus, Ebola virus, bovine viral diarrhea virus, and hepatitis C virus (Stuyver et al., 2003; Reynard et al., 2015; Ehteshami et al., 2017; Urakova et al., 2018; Yoon et al., 2018; Painter et al., 2019; Toots et al., 2019). *In vitro* studies showed that NHC has a significant inhibitory effect on SARS-CoV-1 at EC90 of 6 μM in Vero 76 cells (Barnard et al., 2004). Also, the drug was shown to have a potent antiviral activity (IC50 of 35 nM) against HCoV-NL63 (Pyrc et al., 2006). NHC also efficiently inhibits murine hepatitis virus (a group 2 CoV) and MERS-CoV infections *in vitro* and results in the accumulation of transition mutations with a genetic barrier to resistance (Agostini et al., 2019). Recently, NHC was shown to successfully suppress SARS-CoV-2 *in vitro* with an IC50 of 0.3 μM (Sheahan et al., 2020b). It also exhibited broad-spectrum antiviral activity against SARS-CoV-1, MERS-CoV, and a related bat-CoV *in vitro* (Sheahan et al., 2020b). Moreover, prophylactic and therapeutic treatment with EIDD-2801 enhanced pulmonary function and significantly decreased viral titers in the lungs of SARS- and MERS-CoV–infected mice (Sheahan et al., 2020b). The efficacy of EIDD 2801 is being investigated in randomized clinical trials but no results have been published yet.

#### Oseltamivir

Oseltamivir is an antiviral drug approved for treatment or prophylaxis for both seasonal and pandemic influenza infections caused by influenza A and B viruses (Smith et al., 2011). It blocks the release of influenza progeny viruses and hence restrains the spread of influenza infection in the respiratory tract (Moscona, 2005).

CoVs do not possess a functional homologue to influenza virus neuraminidase (the target of oseltamivir) neuraminidase making it unlikely for oseltamivir to be effective against SARS-CoV-2 (McCreary and Pogue, 2020). Oseltamivir was empirically used with other antivirals to treat patients infected with SARS-CoV-1 but was not shown to be effective (Drosten et al., 2003; Hsu et al., 2003; Nicholls et al., 2003; Poutanen et al., 2003; Sung et al., 2004). Similarly, no inhibitory effect was observed when oseltamivir was tested against SARS-CoV-1 *in vitro* (Tan et al., 2004). In addition, oseltamivir treatment did not induce any improvement in hospitalized patients with MERS-CoV (Al-Tawfiq et al., 2014). During the SARS-CoV-2 pandemic in Wuhan, oseltamivir was frequently used likely because of the concern of influenza and not as targeted therapy for SARS-CoV-2. The drug was empirically used either with or without antibiotics and corticosteroids (Ding et al., 2020; Huang et al., 2020; Wang D. et al., 2020) and was also given to patients diagnosed with both SARS-CoV-2 and influenza virus (Ding et al., 2020). As of yet, there is no *in vitro* or *in vivo* evidence of oseltamivir efficacy *or* activity against SARS-CoV-2. Therefore, the rationale behind the ongoing clinical trials is not clear.

#### Others

A number of other virus direct-acting antivirals are being considered for the treatment of COVID-19 despite the lack of data supporting their antiviral activity against SARS-CoV-2. Penciclovir is a synthetic acyclic guanine derivative that inhibits DNA polymerase and is used for the treatment of herpes simplex infections in including HSV-1 and -2 (Earnshaw et al., 1992; Razonable, 2011; Mondal, 2016). *In silico*, penciclovir seems to interact with the nonstructural protein 12 (nsp12) that possesses the RdRp activity (Dey et al., 2020); yet, *in vitro* studies demonstrated poor antiviral activities against SARS-CoV-2 with EC50 of 95.96 μM (Dhama et al., 2020; Wang M. et al., 2020). Sofosbuvir, a hepatitis C RdRp inhibitor, was predicted *in silico* to tightly bind SARS-CoV-1 and -2 RdRps (Noell et al., 2015; Elfiky, 2020a; Elfiky, 2020b). Azvudine (FNC) is a reverse-transcriptase inhibitor (NRTIs) that possess antiviral activity against HIV and hepatitis B viruses (Wang et al., 2011; Zhou et al., 2012). Despite the lack of any pre-clinical efficacy data and the absence of the drug target in SARS-CoV-2, clinical trials are underway in China to evaluate its effectiveness for moderate to severe COVID-19 pneumonia. Triazavirin, a guanine nucleotide analog, is currently being assessed in a multicenter randomized clinical trial despite that available data is limited to its antiviral activity against influenza viruses (Kiselev et al., 2012; Shvetsov et al., 2018).

Another interesting approach that has been proposed for COVID-19 treatment is the use of decoy ACE2 molecules to block SARS-CoV-2 from binding to their target cells. A clinical-grade human recombinant soluble ACE2 (hrsACE2) showed a significant inhibitory effect of viral growth during the early stages of SARS-CoV-2 infection in Vero E6 cells, human blood vessel organoids, and human kidney organoids expressing ACE-2 receptor (Monteil et al., 2020). However, this approach has not been tested in animal models. The safety and tolerability of APN01 have been demonstrated in phase II trials including healthy volunteers and patients with pulmonary arterial hypertension, acute lung injury, and ALI/ARDS. A phase II randomized clinical trial of hrsACE2 (APN01) has been launched and examined for lung disease during the initial stages of SARS-CoV-2 infection (Harrison, 2020b).

### Host Targeting Agents

#### Azithromycin

Azithromycin (AZM) is an FDA approved broad-spectrum macrolide antibiotic. (Fohner et al., 2017). Besides its antibacterial activity, AZM appears to possess a broad antiviral capacity. *In vitro* studies revealed that AZM inhibits the replication of the Zika virus by targeting a late stage of its lifecycle (Bosseboeuf et al., 2018; Li et al., 2019). AZM also showed anti-rhinovirus activity *in vitro* through stimulating the interferon pathway (Gielen et al., 2010; Schögler et al., 2015). Moreover, AZM possesses antiviral activity against influenza A/H1N1pdm09 virus by interfering with its cell entry. AZM decreased A/H1N1pdm09 virus titers in the lungs and reduced infection-induced hypothermia in mice (Tran et al., 2019). AZM was also effective against the Ebola virus in cell culture but did not show reproducible efficacy in mouse models and was not effective in guinea pig model (Madrid et al., 2015).

Before the emergence of SARS-CoV-2, AZM was not previously tested against CoVs. *In vitro* studies showed that hydroxychloroquine/azithromycin (HCQ/AZM) (5 μM/5 μM) combination significantly diminished SARS-CoV-2 replication (Andreani et al., 2020). However, the used AZM dose is about 5.7 times higher than the therapeutically achieved plasma concentration (Food and Drug Administration, 2010). In France, an open-label non-randomized clinical trial was conducted on 36 COVID-19 patients and showed 100% recovery in patients receiving HCQ/AZM and a significant reduction in viral load in patients receiving HCQ alone. However, the small sample size (only 6 patients in the HCQ/AZM arm) and the non-randomized open-label design of the trial limit its reliability (Gautret et al., 2020). Consistently, a multi-center retrospective observational study from the United States revealed that the administration of HCQ with or without AZM resulted in a significant decrease in mortality in hospitalized COVID-19 patients (Arshad et al., 2020). In contrast, Magagnoli et al. demonstrated that the use of HCQ alone or in combination with AZM has no significant reduction in the need for mechanical ventilation. Also, the administration of HCQ alone was associated with a higher mortality rate (Magagnoli et al., 2020). Several clinical trials, with or without randomization, aimed at investigating the efficacy of HCQ/AZM combination in COVID-19 patients have been registered.

#### Ivermectin

Ivermectin is a broad-spectrum anthelmintic agent (González Canga et al., 2008). It binds to the glutamate-activated chloride channel in the parasites’ nerve and muscle cells resulting in its hyperpolarization, paralysis, and death (Ikeda, 2003). It has a broad antiviral activity spectrum *in vitro*. Ivermectin was shown to inhibit the replication of influenza A viruses, Venezuelan equine encephalitis virus, dengue virus, HIV-1, flavivirus, West Nile virus, and pseudorabies virus (Mastrangelo et al., 2012; Wagstaff et al., 2012; Lundberg et al., 2013; Tay et al., 2013; Götz et al., 2016; Lv et al., 2018; Yang S. N. Y. et al., 2020). A phase III, randomized, double-blind, placebo-controlled clinical trial in Thailand revealed that a once-daily dose for three days is well-tolerated and resulted in enhanced serum clearance of dengue virus non-structural (NS1) protein. (Yamasmith et al., 2018). The broad-spectrum antiviral activity of ivermectin is mainly attributed to its interaction with importin (IMP) α/β1 heterodimer having a role in nuclear import (Caly et al., 2012; Jans et al., 2019). Several studies revealed the role of IMPα/β1 in the nuclear import of SARS-CoV-1 nucleocapsid protein (Rowland et al., 2005; Timani et al., 2005; Wulan et al., 2015). Moreover, SARS-CoV-1 blocks the host’s antiviral response through retaining IMPα/β1 on the rough ER/Golgi membrane, inhibiting STAT1 nuclear transport and antagonizing its antiviral activity (Frieman et al., 2007). Treating Vero cells expressing the human signaling lymphocytic activation molecule (Vero-hSLAM cells) with ivermectin (5 μM) 2 h after SARS-CoV-2 infection resulted in a 5000-fold decrease in viral RNA 48 h post-infection with an IC50 of 2 µM (Caly et al., 2020). However, this concentration is about four times higher than the therapeutically achieved plasma concentration without side effects (Food and Drug Administration, 2009b), suggesting a limited antiviral activity for ivermectin in humans. Randomized clinical trials investigating the efficacy of ivermectin adjuvant to HCQ or CQ are ongoing.

#### Nafamostat and Camostat

Nafamostat mesylate and camostat mesylate are synthetic serine protease inhibitors approved in Japan for the treatment of pancreatitis. Camostat was shown to suppress the replication of influenza A/H1N1pdm09 and A/H3N2 viruses in the primary human tracheal epithelial (HTE) cells. It also reduced the concentrations of the cytokines IL-6 and TNF-alpha in primary cultures (Yamaya et al., 2015). *In vitro* studies revealed that nafamostat possesses antiviral activities against epidemic and zoonotic CoVs including MERS-CoV (Yamamoto et al., 2016). The anti-MERS-CoV activity is achieved through inhibition of the host protease (TMPRSS2) required for fusion-activation of the virus (Gierer et al., 2013). Consistently, Zhou et al. demonstrated that camostat displayed antiviral activity in a pathogenic animal model for SARS-CoV-1 infection by inhibiting the enzymatic activity of TMPRSS2 and other cell-surface proteases involved in CoV activation (Zhou et al., 2015). Recently, camostat was shown to suppress SARS-CoV-2 S protein-initiated fusion in 293FT cells ectopically expressing ACE2 and TMPRSS2 (Hoffmann et al., 2020). Currently, the safety and efficacy of camostat are being assessed in randomized clinical trials. Moreover, other randomized studies comparing the efficacy of camostat-HCQ combination to HCQ-AZM combination or monotherapy are being conducted.

#### Teicoplanin

Teicoplanin, formerly known as teichomycin A, is a natural glycopeptide antibiotic produced from the actinomycete *Actinoplanes teichomyceticusteino* and commonly used for the treatment of gram-positive infections (Parenti et al., 1978; Borghi et al., 1984; Svetitsky et al., 2009). The drug is not approved by the FDA but is commercially available in Europe, Asia, and South America (Parente and Laplante, 2017). The bactericidal activity of the drug is mediated mainly by the inhibition of peptidoglycan synthesis of the bacterial cell wall (Corti et al., 1985; Murray et al., 2015). In addition to its antibacterial activity, teicoplanin is a viral entry inhibitor with a broad-spectrum of activity. Teicoplanin and/or teicoplanin derivatives were shown to have *in vitro* antiviral activities against several viruses such as feline infectious peritonitis virus (FIPV), SARS-CoV-1, HIV-1, influenza virus, flaviviruses, including Ebola virus, and RSV (Balzarini et al., 2006; Preobrazhenskaya and Olsufyeva, 2006; Burghgraeve et al., 2012; Bakai-Bereczki et al., 2014; Wang Y. et al., 2016). Teicoplanin aglycon derivatives were reported to suppress the intracellular replication of the hepatitis C virus *in vitro* (Obeid et al., 2011). Moreover, teicoplanin therapy significantly reduced the viral load in a patient with chronic hepatitis C (Maieron and Kerschner, 2012). Zou et al. reported that teicoplanin suppresses the entry of the Ebola virus, SARS-CoV-1, and MERS-CoV by blocking the activity of cathepsin L in the late endosome/lysosome (Zhou et al., 2016). Inhibition of the cysteine protease, cathepsin L, prevents the cleavage and the subsequent activation of the S glycoprotein of CoVs, a key step required for virus fusion and release of the viral genome (Zhou et al., 2016; Baron et al., 2020). It was recently shown that teicoplanin efficiently restricts the entry of SARS-CoV-2 pseudovirus with an IC50 of 1.66 μM, which is less than the usual trough plasma drug concentration (7–8 µM) (Zhang et al., 2020a). Molecular docking studies show an interaction between teicoplanin and SARS-CoV-2 3CL^Pro^ (Azam, 2020). Further pre-clinical investigations are needed to assess the inhibitory effect of teicoplanin against wildtype SARS-CoV-2.

#### Nitazoxanide

Nitazoxanide is a thiazolide derivative that possesses a broad-spectrum antiviral and anti-parasitic activity (White, 2004; Rossignol, 2014). It is FDA-approved for the treatment of parasitic infections caused by *Cryptosporidium parvum* and *Giardia lamblia* (Fox and Saravolatz, 2005). Nitazoxanide has broad antiviral activity against several DNA and RNA viruses. *In vitro* studies confirmed its effectiveness against RSV (Nichols et al., 2008), HIV (Rossignol, 2014), Japanese encephalitis virus (Shi et al., 2014), hepatitis B and C viruses (Korba et al., 2008), rotavirus (La Frazia et al., 2013), and Ebola virus (Jasenosky et al., 2019). Also, nitazoxanide is effective against multiple anti-influenza virus types/subtypes. It inhibits hemagglutinin maturation and incorporation in the cell surface by blocking its post-translational glycosylation (Rossignol, 2014). Previous *in vitro* studies showed that it has robust antiviral activity against murine CoV and MERS-CoV by blocking viral entry and replication (Cao et al., 2015; Rossignol, 2016). The drug inhibited SARS-CoV-2 replication in Vero E6 cells at a low concentration (EC50 = 2.12 μM) (Wang M. et al., 2020) and this concentration is three times lower than the common therapeutically achieved one (Fox and Saravolatz, 2005). In addition, to its host-acting antiviral activity, nitazoxanide inhibits the production of pro-inflammatory cytokines, including interleukin 6 (IL-6), a property that can be very beneficial in COVID-19 treatment (Russell B. et al., 2020). Clinical trials, including randomized ones, investigating the efficacy and safety of nitazoxanide are ongoing.

### Drugs with Mixed Modes of Action

#### Umifenovir

Umifenovir (commonly known as arbidol) is an antiviral medication licensed in Russia and China for prophylaxis and treatment of influenza virus infections. It has shown a broad-spectrum antiviral activity *in vitro* and *in vivo* against a wide range of enveloped or non-enveloped RNA or DNA viruses, including members of the families *Orthomyxoviridae, Paramyxoviridae, Flaviviridae, Filoviridae, Herpesviridae Picornaviridae*, and *Coronaviridae* (Leneva et al., 2002; Pécheur et al., 2016; Haviernik et al., 2018; Herod et al., 2019). Umifenovir has been shown to possess both direct-acting and host-targeting antiviral activities through a range of proposed modes of action including interacting with host membrane lipids and proteins and viral glycoproteins, elevating the endosomal pH, and interfering with intracellular trafficking (reviewed in details in (Blaising et al., 2014)).

Umifenovir was shown to inhibit SARS-CoV-1 infection in GMK-AH-1 cells (Khamitov et al., 2008). It also efficiently inhibited SARS-CoV-2 infection *in vitro* at a low concentration (4.11 µM) suggesting that it could be effective in the treatment of COVID-19 (Wang X. et al., 2020). A retrospective case-control study was conducted in Wuhan, China, found that umifenovir was more effective than oseltamivir as post-exposure prophylaxis among healthcare workers and family members with high exposure risk to COVID-19. The study had some limitations, including the use of oseltamivir as a comparator drug (Zhang et al., 2020c). Wang et al. reported clinical improvement in three COVID-19 patients treated with a combination of lopinavir/ritonavir, umifenovir, and traditional Chinese Medicine (Wang et al., 2020a). Deng et al. showed in a retrospective observational study that 16 COVID-19 patients treated with umifenovir plus lopinavir/ritonavir combination had more favorable viral clearance and chest CT findings compared to lopinavir/ritonavir monotherapy (Deng et al., 2020). A retrospective study demonstrated that early administration of umifenovir or umifenovir in combination IFN significantly shortened the duration of viral shedding in COVID-19 patients (Zhou Y. et al., 2020). Furthermore, a limited retrospective cohort study reported that umifenovir tends to improve the discharging rate and reduce mortality rate (Wang et al., 2020b). Another retrospective study on family members and healthcare workers showed that umifenovir could act as protective post-exposure prophylaxis of COVID-19 transmission (Zhang et al., 2020b). Reconcilable results were observed in a retrospective case-control study that found a preventative role of prophylactic umifenovir use in lowering the incidence of SARS-CoV-2 infection of health professionals but no correlation to hospitalization rate was found (Yang C. et al., 2020).

In contrast, a clinical study (NCT04252885) involving 44 mild-to-moderate COVID-19 patients, found no clinical benefit for umifenovir or lopinavir/ritonavir monotherapy compared to the control standard-of-care group (Li Y. et al., 2020). Similarly, another retrospective study indicated that umifenovir treatment in mild-to-moderate COVID-19 patients was neither with improvement of clinical symptoms nor altered the duration to viral clearance (Jun et al., 2020). Despite the lack of evidence supporting its efficacy, umifenovir was added to the latest version of the Guidelines for the Prevention, Diagnosis, and Treatment of Novel Coronavirus-induced Pneumonia issued by the National Health Commission (NHC) China for tentative treatment of COVID-19 (Dong et al., 2020).

#### Chloroquine Phosphate and Hydroxychloroquine

Chloroquine phosphate (CQ), a 4-aminoquinoline compound, is approved for the treatment of malaria and extraintestinal amebiasis (Food and Drug Administration, 2009a). It is also being used as an off-label anti-inflammatory agent for the treatment of rheumatoid arthritis and lupus erythematosus owing to its immunomodulatory properties (National Institute of Diabetes and Digestive and Kidney Diseases, 2012). Hydroxychloroquine (HCQ), a chloroquine phosphate derivative, is also an FDA-approved antimalarial and immunomodulatory agent. It is prescribed for the prophylaxis and treatment of uncomplicated chloroquine-sensitive malaria, treatment of chronic discoid lupus erythematosus, systemic lupus erythematosus, and acute and chronic rheumatoid arthritis in adults (Food and Drug Administration, 2018).

CQ and HCQ exert their antimalarial activity by accumulating in the parasites’ acidic food vacuoles and inhibiting the transformation of the toxic heme into hemozoin biocrystal that is needed for survival (Parhizgar and Tahghighi, 2017; Food and Drug Administration, 2018). Their anti-rheumatic activity is achieved by hindering the auto-immune response. CQ and HCQ are lysosomotropic agents that gain access into the lysosomes and increase their pH leading to the inhibition of acidic proteases. This process interferes with the antigen processing, dimerization of the major histocompatibility complex (MHC) class II, antigen presentation, and the stimulation of CD4+ T cells. Furthermore, they diminish the production of pro-inflammatory cytokines by interfering with Toll-like receptor (TLR) signaling (in the endosome) and inhibiting the nucleic acid sensor cyclic GMP-AMP (cGAMP) synthase (in the cytosol) (Savarino et al., 2003).

Besides their antimalarial and immune-modulatory effects, CQ and HCQ possess antiviral activities. Upon uptake, HCQ/CQ get trapped inside endosomes, lysosomes and Golgi vesicles (mammals) increasing its PH (Al-Bari, 2015). In this context, CQ and HCQ may be potential treatment options for viruses depending on low endosomal pH for cellular internalization (Al-Bari, 2017). They possess promising activity against human HIV-1. However, its exact mechanism of action is still not fully understood. Still, it is likely attributed to the impairment of the post-translational processing of HIV glycoproteins by increasing the pH of the trans-Golgi network (Tsai et al., 1990; Romanelli et al., 2004; Paton et al., 2012). Moreover, CQ inhibits the replication of human influenza A viruses (H1N1 and H3N2) *in vitro* (Ooi et al., 2006) but not *in vivo* (Vigerust and McCullers, 2007). CQ was also tested against dengue virus; *in vitro* studies revealed a decrease in virus titer (Farias et al., 2013), and *in vivo* studies using nonhuman primates (monkeys) showed improvement in symptoms upon treatment with CQ (Farias et al., 2015). However, clinical studies showed that CQ did not reduce the duration of dengue infection and time to recovery, despite improvement in the quality of life of patients (Tricou et al., 2010; Borges et al., 2013). Wang et al. reported that HCQ inhibits dengue virus by the activation of reactive oxygen species and a mitochondrial antiviral signaling protein-mediated host interferon type I antiviral pathway (Wang et al., 2015). CQ also possesses antiviral efficacy against the Zika virus in different cell lines (Delvecchio et al., 2016) and mice models (Li et al., 2017; Shiryaev et al., 2017). It blocks the release of the Zika virus from the endosome and inhibits virus-induced autophagy and autophagy-dependent replication (Zhang et al., 2019). CQ was also shown to inhibit the Japanese encephalitis virus by restricting its pH-dependent internalization in rat neuroblastoma cells (Zhu et al., 2012).

The efficacy of CQ and HQC against SARS-CoV-1, MERS-CoV, and other human CoVs has been also described (Keyaerts et al., 2004; Vincent et al., 2005; Kono et al., 2008; de Wilde et al., 2014). CQ possesses potent *in vitro* antiviral activity against SARS-CoV-1 when added pre- or post-infection. This antiviral activity was partially attributed to the impairment of ACE2 terminal glycosylation that is needed for SARS-CoV-1 binding (Vincent et al., 2005). CQ-induced increase in endosomal pH inhibits the pH-dependent SARS-CoV-1 internalization (Yang et al., 2004). CQ increased the survival of newborn mice infected with HCoV-OC43 (Keyaerts et al., 2009). However, it did not result in a significant reduction in SARS-CoV-1 lung virus titer in BALB/c mice (Barnard et al., 2006b).

The antiviral activity of CQ and HCQ against SARS-CoV-2 has also been reported. *In vitro* studies showed that CQ effectively inhibits SARS-CoV-2 in Vero E6 cells (EC50 = 1.13 μM) (Wang M. et al., 2020). Liu et al. demonstrated that CQ has a lower EC50 compared to HCQ (Liu J. et al., 2020). In contrast, Yao et al. demonstrated that HCQ (EC50 = 0.72 μM) exhibits a stronger anti-SARS-CoV-2 activity than CQ (EC50 = 5.74 μM) (Yao et al., 2020). The efficacy of HCQ against SARS-CoV-2 at plasma concentrations that can be obtained using clinically approved doses was tested, but unfortunately, HCQ (1 or 2 μg/mL) did not result in any significant reduction in viral loads in Vero E6 cells (Kang et al., 2020). Preliminary data from more than 100 patients recruited in clinical trials in China demonstrated that CQ was superior to control in ameliorating the symptoms, diminishing the duration of the disease, and achieving total recovery in patients with COVID-19–associated pneumonia (Gao et al., 2020). However, the full results of these studies have not been released yet. Arshad et al. reported in a multi-center retrospective observational study that HCQ treatment resulted in a reduction of COVID-19 mortality compared to no treatment (Arshad et al., 2020). On the contrary, HCQ administration was not associated with significant reductions in the risk of intubation and deaths in two observational studies including COVID-19 hospitalized patients in the United States (Geleris et al., 2020, 19) (Magagnoli et al., 2020). Compelling evidence came from the RECOVERY and Solidarity randomized clinical trials, which recently announced stopping the HCQ arm due to lack of clinical benefit revealed in the interim analysis (Horby and Landray, 2020a; World Health Organization, 2020d). In the RECOVERY trial, 1542 patients randomly received HCQ and compared with 3132 patients who received standard of care alone. The 28-day mortality for the HCQ was 25.7% compared to 23.5% in the control group (hazard ratio 1.11 [0.98–1.26]; p=0.10). Full data from both studies have not been disclosed yet. In acute viral infections administering the antiviral drug early during infection might be of greater clinical benefit. Nonetheless, two randomized clinical trials showed that early administration of HCQ for adult outpatients with mild COVID-19 did not have clinical benefit in terms of clinical recovery and reducing disease severity or viral load (Mitjà et al., 2020; Skipper et al., 2020). Moreover, HCW was not effective in preventing laboratory-confirmed or clinically suspected COVID-19 compared to placebo (Boulware et al., 2020). In conclusion, CQ or HCQ are evidently of no clinical benefit in COVID-19 patients. Moreover, their use for patients with severe COVID-19 was associated with concerns regarding the elongation of the QT interval requiring careful monitoring of the treated patients (Borba et al., 2020; Chorin et al., 2020).

### Disease Modulating Adjunctive Therapy

#### Immunomodulatory Agents

##### Fingolimod

Fingolimod (FTY720) is a sphingosine-1-phosphate (S1P) receptor modulator (Blanc et al., 2015). It induces immunomodulatory properties that trap naïve and memory T cells in lymph nodes resulting in a reduced peripheral circulation (Pfender et al., 2015). It is licensed for the treatment of patients with relapsing multiple sclerosis. (Blanc et al., 2015). *In vitro* and *in vivo* studies suggested that fingolimod can be utilized as a treatment against HIV remission by limiting the viral persistence and its T cells reservoir (Geffin et al., 2017; Pino et al., 2019). The immunomodulatory properties of fingolimod can be useful in suppressing the hyperinflammatory response in severe COVID-19 patients. There were recent reports of COVID-19 patients under fingolimod treatment for multiple sclerosis (Foerch et al., 2020; Merad and Martin, 2020). One patient (a 42-year-old female) had a favorable outcome, while the other (a 57-year old female) required ICU admission and ventilation. Therefore, the timing of fingolimod administration might be critical as its immunosuppressive properties might enhance virus replication when administered early during infection. It might be also important to test patients for varicella-zoster virus infections as there have been reports of relapse in patients treated with fingolimod (Pfender et al., 2015; Manni et al., 2017; Redelman-Sidi et al., 2018). Two clinical trials, including one randomized, were registered to test the effectiveness of fingolimod against COVID-19.

##### Thymosin α1

Thymosin α1 is a naturally occurring 28-amino acid peptide with immunomodulatory properties that can trigger lymphocyte maturation, enhance T cell activation, and improve the immune response (Low and Goldstein, 1984). It has an antineoplastic activity and is used as an anticancer treatment (Billich, 2002; Maio et al., 2010). It was used as an adjuvant with influenza (H1N1pdm09 and H9N2) vaccines to boost the immune response and the vaccine efficacy (Carraro et al., 2012; Wang C. et al., 2016). It is also approved in some countries for the treatment of chronic viral infections such as hepatitis B and C (Andreone et al., 2001; Kullavanuaya et al., 2001; Iino et al., 2005). Moreover, thymosin α1 successfully enhanced cellular immunity against cytomegalovirus disease and HIV (Chadwick et al., 2003; Ji et al., 2007). The drug was patented for the treatment of SARS-CoV-1 infection (Rudolph and Tuthill, 2010). A recent retrospective study evaluated the clinical efficacy and benefit of thymosin α1 for severe COVID-19 patients; thymosin treatment was associated with a significant reduction in mortality. Besides, thymosin α1 boosts the immune functions in patients with severe lymphocytopenia *via* promoting thymus output (Liu Y. et al., 2020). It is currently used in three COVID-19 clinical trials in China with various combinations including PD-1 blocking antibody, recombinant human IFN-α-1b, or protease inhibitors (Harrison, 2020a).

##### Tocilizumab

Tocilizumab is a recombinant anti-human interleukin-6 receptor (IL-6R) monoclonal antibody indicated for the treatment of rheumatoid arthritis (RA), juvenile idiopathic arthritis (JIA), and giant cell arteritis (Woodrick and Ruderman, 2011; Stone et al., 2017). It is also approved for cytokine release syndrome, a side-effect of CAR-T cell therapies (Food and Drug Administration, 2019). IL-6 is crucial for the host to mount an immune response against a wide range of viruses including influenza, vaccinia virus, Andes virus, vesicular stomatitis virus, etc. (Angulo et al., 2017; Velazquez-Salinas et al., 2019).

Early studies on SARS-CoV-1 and MERS-CoV infections demonstrated that an increase in serum pro-inflammatory cytokines such as IL-1B, IL-6, and IL-12, among others, was associated with extensive lung damage in patients (Wong et al., 2004; Huang et al., 2005; Mahallawi et al., 2018). With SARS-CoV-2, respiratory failure from acute respiratory distress syndrome (ARDS) is the leading cause of mortality, with IL-6 levels being a significant predictor (Ruan et al., 2020). The cytokine profile observed in patients with severe COVID-19 is suggestive of cytokine storm, characterized by fatal hypercytokinemia and multi-organ failure (Huang et al., 2020). Even though hyper-inflammation exacerbates lung injury, corticosteroids are not recommended for COVID-19 routine treatment and might worsen respiratory symptoms (Russell C. D. et al., 2020). However, in cases where patients with severe COVID-19 present with “cytokine storm,” immunosuppression with tocilizumab has been proposed as a potential treatment (Mehta et al., 2020). T cell exhaustion was detected in 75% of non-ICU COVID-19 patients and 90% of ICU patients. The reduction in T cell functionality was attributed to the hypercytokinemia, suggesting a potential role for tocilizumab in restoring T cell counts in COVID-19 patients (Diao et al., 2020). A retrospective assessment of tocilizumab treatment found that it can ameliorate and reduce inflammatory biomarker levels supporting its effectiveness in relieving cytokine storms (Luo et al., 2020). A small observational study of patients with severe and critical COVID-19 did not report any mortality upon tocilizumab treatment and showed improvement in patients’ symptoms, hypoxygenemia, and CT lung (Xu et al., 2020). Retrospective analysis of 15 moderate-to-critically ill patients found that tocilizumab treatment was associated with a reduction in IL-6 and CRP levels (an inflammatory activity marker) compared to before initiating treatment (Guaraldi et al., 2020). Another retrospective cohort study found that tocilizumab administration significantly shortened the duration of vasopressor support in hypoxic COVID-19 patients compared to the no tocilizumab control group (2 vs. 5 days, respectively; *p = 0.039*). The time to clinical improvement and invasive ventilation was shorter, albeit not significantly, in the tocilizumab group compared to the control group (Kewan et al., 2020). Interestingly, a COVID-19 patient with multiple myeloma was successfully treated with tocilizumab (Zhang X. et al., 2020).

In late April, a preliminary report of phase II randomized trial on tocilizumab (NCT04331808) described no clear benefit and improvement of clinical outcomes in patients with moderate to severe pneumonia associated with COVID19, yet it showed minor positive trends with critical COVID-19 cases (Regeneron Pharmaceuticals. Inc, 2020). However, the phase III trial was shut down as tocilizumab failed to show clinical benefit in critically ill, mechanically ventilated patients. Thus, further research and evidence on tocilizumab effectiveness and side effects are needed before issuing a treatment recommendation. Tocilizumab is currently being investigated in several trials, including randomized ones, in patients with COVID-19 pneumonia and elevated IL-6.

##### Bevacizumab

Bevacizumab, a humanized monoclonal antibody against the angiogenic vascular endothelial growth factor (VEGF), is being investigated for the treatment of acute lung injury in COVID-19. The literature is conflicting regarding the role of VEGF in the normal lung. Some studies have suggested a protective role for VEGF on the alveolar epithelium following injury (Medford and Millar, 2006). This is supported by the fact that VEGF is found in substantial quantities in the normal lung. Conversely, other studies have proposed that it may participate in the development of non-cardiogenic pulmonary edema, worsening lung injury. This is supported by experiments showing that the administration of soluble VEGF receptors reduces the extent of pulmonary edema (Kaner et al., 2000). A unifying model is that VEGF functions in an autocrine fashion to repair and regenerate the alveolar epithelial surface, yet contributes to the development of pulmonary edema if the alveolar-capillary membrane is disrupted, as is the case during acute lung injury (Medford and Millar, 2006). These findings triggered interest in the potential therapeutic role for bevacizumab in ARDS, a leading cause of COVID-19 mortality. Retrospective studies have identified elevated levels of VEGF in the blood of COVID-19 patients (Huang et al., 2020). Rising VEGF levels were hypothesized to be due to hypoxia and severe inflammation, and thought to be one of the causes of respiratory failure in COVID-19 (Viveiros Rosa and Santos, 2020). Clinical trials, including randomized ones, are underway to test bevacizumab as a treatment for ARDS in COVID-19 patients.

##### Colchicine

Colchicine is an FDA-approved drug for the prophylaxis and treatment of gout flares and the treatment of Familial Mediterranean Fever (FMF). It has other off-label uses, including the treatment of Behcet’s syndrome, an autoimmune vasculitis. The exact mechanism of action of colchicine is not well understood. Its primary mode of action is thought to be tubulin disruption, which leads to the down-regulation of multiple inflammatory pathways (Leung et al., 2015). By preventing microtubule assembly, colchicine disrupts the inflammasome complex and consequently blocks activation of IL-1. It also inhibits microtubule-dependent chemotaxis of neutrophils, generation of leukotrienes and cytokines, phagocytosis, and the (TNF-α)-induced NF-κB pathway. In addition, it has anti-fibrotic effects (Dalbeth et al., 2014; Leung et al., 2015). These properties have led researchers to suggest a potential role for colchicine in the treatment of COVID-19 pneumonia, especially with regards to attenuating the cytokine storm and associated pulmonary edema. A randomized, open-label study found that hospitalized COVID-19 patients in the colchicine group had significantly improved time to clinical deterioration compared to those receiving standard care. Nevertheless, there were no significant differences between the groups when examining cardiac and inflammatory biomarkers (Deftereos et al., 2020). The study was underpowered with limited sample size, but it supports the potential therapeutic benefit of colchicine in COVID-19 patients. Several randomized clinical trials are ongoing to study the safety and efficacy of colchicine to reduce lung complications related to COVID-19.

#### Corticosteroids

##### Methylprednisolone

Methylprednisolone is an FDA-approved immunosuppressive and anti-inflammatory agent. It is indicated for use as a replacement therapy or systemic symptomatic treatment (Yasir et al., 2020). Owing to its anti-inflammatory benefits, at low doses, corticosteroids including methylprednisolone have been widely tested for a variety of viral infections. However, its clinical benefits remain controversial.

In RSV-infected children, no clinical benefits were achieved with the use of corticosteroids (Corneli et al., 2007). Moreover, the use of corticosteroids appeared to be harmful in viral hepatitis and cerebral malaria (McGee and Hirschmann, 2008). A meta-analysis of 6548 patients from ten studies comparing the effects of corticosteroids, including methylprednisolone, to placebo in patients with influenza-associated pneumonia revealed that corticosteroids were associated with increased mortality (Ni et al., 2019).

An uncontrolled, non-randomized study of high-dose methylprednisolone treatment found that the majority (89%) of SARS patients recovered from progressive lung disease one-week post-illness (Sung et al., 2004). However, a systematic review of corticosteroids use in SARS revealed that 25 studies were inconclusive, while four revealed evidence of possible harm including decreased virus clearance and drug-induced complications (Stockman et al., 2006). Corticosteroids were commonly utilized in the treatment of MERS-CoV–infected patients but its effect on clinical outcome was controversial. Thus, a multicenter study was conducted to investigate the effectiveness of corticosteroid therapy on MERS-associated mortality and viral clearance. This study showed that corticosteroid therapy did not improve survival and was associated with a delay in MERS-CoV clearance (Arabi et al., 2017). Although clinical evidence from other viral infections is not supportive of clinical benefit, several clinical trials including a randomized one, are underway to assess the safety and efficacy of methylprednisolone in COVID-19.

##### Dexamethasone

Dexamethasone is a steroid that has been indicated to treat a wide range of conditions including asthma, allergies, and autoimmune conditions such as lupus and rheumatoid arthritis (Shrestha et al., 2019; Elkharwili et al., 2020; Wang Q. et al., 2020). It has been used for its anti-inflammatory and immunosuppressant effects. While not a COVID-19 cure, dexamethasone has emerged with strong evidence of improving COVID-19 survival and mortality in severely ill patients. Preliminary results released from a randomized, controlled, Phase II/III clinical trial found that dexamethasone significantly reduced deaths among those with severe COVID-19 illnesses. Of the total 11,320 patients included in the RECOVERY trial, 2104 patients were randomized to receive dexamethasone for 10 days and 4321 were randomized to usual care only. The remainder were randomized to one of the other treatment arms. Compared to standard care, dexamethasone was found to significantly reduce deaths by one-third (29.3% vs. 41.4%; rate ratio, 0.64 [0.51 to 0.81]) among ventilated patients and by one-fifth (23.3% vs. 26.2%; rate ratio, 0.82 [0.72 to 0.94]) in patients who require oxygen only. Although the drug was successful in treating many seriously ill patients, there was no benefit in administering it to those with milder cases who did not require respiratory support at randomization (The RECOVERY Collaborative Group, 2020). Additional 14 clinical trials are planned to investigate the efficacy of dexamethasone against COVID-19 with eight trials expected to complete in 2020.

#### Convalescent Plasma

The use of antibodies from recovered COVID-19 patients is currently considered to treat active cases. Antibodies provide passive immunity by viral neutralization or possibly by inducing antibody-dependent cellular cytotoxicity mechanism (Casadevall and Pirofski, 2020). A fast, though not very scalable, way to provide passive immunization is by using convalescent plasma from recovered COVID-19 patients. The use of convalescent sera has shown promise in various infectious diseases including measles, poliomyelitis, mumps, and influenza (Casadevall and Pirofski, 2020). Convalescent plasma therapy was also evaluated during the SARS-CoV-1 outbreak in 2003, resulting in a significantly higher 22-day discharge rate among treated patients compared to controls (Cheng et al., 2005). Nonetheless, other studies on the use of convalescent plasma to treat SARS patients were inconclusive (Stockman et al., 2006). During the MERS outbreak in South Korea, two of three MERS patients with respiratory failure who received convalescent plasma infusion demonstrated neutralizing antibody activity (Ko et al., 2018).

Based on these previous experiences, the FDA facilitated access to COVID-19 convalescent plasma for compassionate use in patients with immediately-life threatening COVID-19 under emergency use authorization (EUA) (Food and Drug Administration, 2020). A study from the USA reported encouraging preliminary results on the safety and efficacy of convalescent plasma therapy in patients with life-threatening COVID-19 disease. At least one-point clinical improvement was demonstrated in 36% of patients on day 7, and 76% had improved or been discharged by day 14. No adverse events were observed indicating convalescent plasma is a safe treatment option for severely ill COVID-19 patients (Salazar et al., 2020). These findings were consistent with another study of 5000 patients treated with convalescent plasma. The incidence of serious adverse events was <1% during the first four post-infusion and the overall seven-day incidence of mortality was 14.9% (Joyner et al., 2020). Liu et al. assessed the outcomes of convalescent plasma in 39 severely ill COVID-19 patients. Compared to matched control, convalescent plasma administration significantly improved survival rate in non-intubated patients but not in intubated ones (Liu S. et al., 2020). Data from an open-label, multicenter, randomized clinical trial of 103 COVID-19 patients with a severe or life-threatening disease show clinical improvement within 28 days in 52% (n=27) of patients who received convalescent plasma compared to 43% (n=22) of patients in the control group. However, there was no difference in the 28-day mortality rate between both groups. The negative viral PCR conversion rate was higher (87.2%) in the treatment group than the standard care group (37.5%) (Li L. et al., 2020). Unfortunately, the study was terminated early as the outbreak was waning in China. A number of clinical trials including randomized ones have already been launched and others were submitted to the FDA to test the effect of convalescent plasma as a treatment for COVID-19.

## Conclusion

The COVID-19 pandemic constitutes an unprecedented challenge to global public health. The first wave of the pandemic was largely mitigated thanks to worldwide lockdowns that averted millions of infections (Hsiang et al., 2020). The world is counting on the development of vaccines to effectively control the pandemic; however, while early clinical trials revealed promising results, a vaccine is unlikely to be widely available before late 2021 (Funk et al., 2020). Thus, the repurposing of existing drugs is the fastest approach to mitigate the COVID-19 burden for the near future. The rapid response to undertake clinical trials of repurposed drugs initially promised fast results; however, most of the early have been disappointing or controversial, to say the least. Several clinical trials are ongoing worldwide and some have already been concluded. These efforts have successfully proven clinical and survival benefits for remdesivir and dexamethasone in hospitalized patients with severe COVID-19. It remains important to identify drugs that can be safely used during the early stage of infection to prevent progression to severe disease and deaths.

## Author Contributions

HZ conceived the idea and supervised the writing. HZ, MK, and MA developed the review outline. MK and MA led and coordinated the drafting of the manuscript. MK, MA, GH, NS, AA, HA, and SH wrote the manuscript. AE critically reviewed the manuscript. All authors contributed to the article and approved the submitted version.

## Funding

This paper was supported by an American University of Beirut, Faculty of Medicine MPP (grant number 320126) and the Lebanese National Council for Scientific Research (CNRS; grant number 103937) to HZ.

## Conflict of Interest

The authors declare that the research was conducted in the absence of any commercial or financial relationships that could be construed as a potential conflict of interest.
